# Our New York Letter

**Published:** 1888-05

**Authors:** 


### Correspondence.

### OUR NEW YORK LETTER.

DR. HAMMOND ON "THE SUPERIORITY OF HANGING AS A MODE OF EXECUTION"—DR. AUSTIN FLINT'S MEMORY HONORED—THE CARTWRIGHT LECTURES—THE PATHOLOGY OF FEVER—A NEW EYE AND EAR HOSPITAL.

*Editors Atlanta Medical and Surgical Journal:*

At a recent gathering of the members of the Society of Medical Jurisprudence and State Medicine, Dr. Wm. A. Hammond presented a paper on "The Superiority of Hanging as a Method of Execution." In early times, he said, among the Jews, strangulation from a rope around the criminal's neck was the custom, and the same method was used by the Egyptians, Persians and other nations with but some slight modifications as to detail. No attempt was made to dislocate the neck until modern times. Dr. Hammond declared that by breaking the neck the agony was by no means lessened, and that death was produced just as effectually by strangulation alone, being a perfectly painless operation if the rope is applied properly. He attributed the frequent failures in causing speedy death to want of skill on the executioner's part. In the case of a man who was revived, after hanging for half a minute and pardoned, there was no pain felt, and experiments had verified this, the sensations produced being general tingling over the surface of the body, noise in the ears and flashes of light, but no real pain. The convulsion of the face and limbs, he said, did not indicate pain, but were the same symptoms as occurred in epilepsy. If the criminal was placed in a chair, the rope being accurately adjusted to the neck, and the subject hauled up by the aid of a pulley, painless death, he said, would occur in seven or eight minutes. In the discussion of the paper, Dr. N. E. Brill stated that the rope, in his opinion, would not produce enough congestion of the brain to destroy sensation.

Dr. Hammond thereupon offered to prove the contrary by an experiment upon the person of Dr. Brill, which was agreed to, and he submitted to having his carotids compressed with much stoicism. He, however, would not admit that the pressure caused abolition of sensation, and expressed himself in favor of the guillotine, and also of electricity, if it could be properly applied. Other gentlemen present considered hanging the best means of execution; but some opposed capital punishment entirely. At the end of the meeting a resolution was passed protesting against the use of electricity as a means of inflicting the death penalty.

A large gathering of the medical profession took place a few evenings ago, in the Carnegie Laboratory, for the purpose of honoring the memory of the deceased Dr. Austin Flint. This building is in East 26th street, and belongs to the Bellevue Hospital Medical College, the Alumni Association of which has erected a memorial tablet to Dr. Flint. The exercises were presided over by Dr. David Webster, and many prominent physicians of the college faculty and others were present. The presentation of the tablet was made by Dr. Leroy Milton Yale, who gave an interesting description of the life and work of Dr. Flint, and said that his enthusiasm never became dormant, and even age could not diminish his desire to gain knowledge. At the termination of this eulogy the memorial tablet was unveiled. The tablet is made of bronze and bears the inscription: "Austin Flint, M. D., LL. D. Born October 20, 1812. Died March 13, 1886. Erected by the Bellevue Hospital Medical College Alumni Association." The address of acceptation was then made by Dr. Isaac E. Taylor, who is president of the faculty.

This year the Cartwright lectures, under the auspices of the Alumni Association of the College of Physicians and Surgeons, are delivered by Wm. H. Welch, M. D., Professor of Pathology in John Hopkins University, Baltimore, his subject being, "The General Pathology of Fever." Dr. Welch is an old New Yorker and his first lecture was well attended. I noticed that the audience was largely made up of young physicians, many of whom were personal friends of the lecturer. The speaker defined fever as a complexity of symptoms, of which increased temperature is the most prominent, but not necessarily the cause of the others. In considering how rise of temperature is produced, he drew attention to heat production, heat loss and the regulating mechanism which balances the two so that the internal temperature remains constant. He said experiments showed that, although the heat production in an animal in fever is greater than that of one under like conditions of nourishment, it is, as a rule, less than that of the same animal upon a full diet. Heat energy of the body was the result of chemical changes in its proteids, fats and carbohydrates. Numerous experiments were described to prove that increased oxidation is a part of the fever process, and also that there is an increased production of heat in fever. One important conclusion was that while an individual in fever produced more heat than he would in health under similar conditions as to food and muscular exercise, he does not necessarily produce more heat in fever than he would in health on a full diet. A man in health may produce twice the quantity of heat he does in fever, and it is impossible to explain febrile rise of temperature simply on the basis of increased thermogenesis. It is the mechanism, he said, which controls the relation of heat production to heat loss, which is primarily disturbed in fever. This he believed to be a nervous mechanism. Nervous impulses could control chemical changes which would result in the production of heat, independently of visible physical alterations in the tissues. Numerous experiments were quoted to verify that there are nerves controlling heat production in muscles distinct from motor nerves. There are also regions in the central nervous system which appear in some way connected with those nerves and through them control the chemical processes which produce heat. He thought the pyrogenic agent must consequently act in some way upon the heat regulating mechanism. Dr.

Welch favored the neurotic doctrine of fever, which holds that we may have a fever of purely nervous origin without a pyrogenic agent in the blood.

The certificate of incorporation was filed this week for the new Amsterdam Eye and Ear Hospital, which is to be a free institution with dispensary attached. There will also be connected with it a school where ophthalmology and otology will be taught. Among the trustees are Drs. John R. Pooley, L. Bolton Bangs, Mr. J. Romaine Brown and several others.

A. F.

*New York, April 8, 1888.*

---

IDIOSYNCRASY TO ANTIPYRIN.—A somewhat similar case to that lately recorded by Dr. Sturge recently came under my notice. I administered to a lady on two different occasions eight grains of antipyrin for attacks of migraine, and on each occasion, very shortly after taking it, a tight feeling of constriction was felt across the chest, with a burning sensation in the pharynx. These symptoms were immediately followed by sneezing, by intense suffusion of the eyes, and by quantities of mucus flowing from the nose, giving her all the appearances of having a severe attack of coryza; there was also great irritation in the larynx, causing severe fits of coughing, but unattended with expectoration. After a quarter of an hour these uncomfortable symptoms gradually subsided. There was no urticaria. I followed it up on each occasion with an equivalent dose of antifebrin (3 grains) which (with one repetition in the course of an hour on the first occasion, but which was not required on the second) completely relieved the severe hemicrania, as it has done on subsequent trials without using antipyrin at all. It appears, therefore, that antifebrin may be used equally with antipyrin in migraine as in febrile conditions, and may replace it with advantage where the latter disagrees.—*H. Coupland Taylor, M. D., in British Medical Journal, Orotava, Teneriffe.*

